# Regenerative Inflammation: Lessons from *Drosophila* Intestinal Epithelium in Health and Disease

**DOI:** 10.3390/pathogens2020209

**Published:** 2013-04-02

**Authors:** Stavria Panayidou, Yiorgos Apidianakis

**Affiliations:** Department of Biological Sciences, University of Cyprus, Nicosia 1678, Cyprus; E-Mails: spanay06@ucy.ac.cy (S.P.); apidiana@ucy.ac.cy (Y.A.)

**Keywords:** *Drosophila*, innate immunity, inflammation, cancer, regeneration, intestine

## Abstract

Intestinal inflammation is widely recognized as a pivotal player in health and disease. Defined cytologically as the infiltration of leukocytes in the lamina propria layer of the intestine, it can damage the epithelium and, on a chronic basis, induce inflammatory bowel disease and potentially cancer. The current view thus dictates that blood cell infiltration is the instigator of intestinal inflammation and tumor-promoting inflammation. This is based partially on work in humans and mice showing that intestinal damage during microbially mediated inflammation activates phagocytic cells and lymphocytes that secrete inflammatory signals promoting tissue damage and tumorigenesis. Nevertheless, extensive parallel work in the *Drosophila* midgut shows that intestinal epithelium damage induces inflammatory signals and growth factors acting mainly in a paracrine manner to induce intestinal stem cell proliferation and tumor formation when genetically predisposed. This is accomplished without any apparent need to involve *Drosophila* hemocytes. Therefore, recent work on *Drosophila* host defense to infection by expanding its main focus on systemic immunity signaling pathways to include the study of organ homeostasis in health and disease shapes a new notion that epithelially emanating cytokines and growth factors can directly act on the intestinal stem cell niche to promote “regenerative inflammation” and potentially cancer.

## 1. Introduction

Inflammation is the physiologic response to tissue injury or infection. In its acute form, it is vital for tissue repair, homeostasis reinstatement, and organism survival. The medical hallmarks of inflammation are: pain (dolor); redness (rubor); heat (calor), which refers to an increase in temperature due to vascular dilatation and delivery of warm blood to the area of the tissue damage; swelling (tumor), *i.e.*, fluid accumulation in the extravascular space and the migration of the inflammatory cells into the area; and, loss of function (functio laesa). Inflammation can become systemic, thus affecting the whole body rather than only one part of it. At the cellular level, inflammation involves the activation of tissue-specific (e.g., mast cells for the intestinal epithelium) and non-tissue-specific (e.g., macrophages, neutrophils, dendritic cells, T-cells, B-cells) cell types of the immune system [1]. Macrophages and neutrophils are the first line of immunity against invading pathogens [1]. However, if tissue homeostasis is perturbed, these cells release soluble factors, including cytokines and chemokines, in order to attract additional leukocytes into the site of damage [1]. Nevertheless, in chronic inflammation, the chronically perturbed tissue homeostasis creates a condition of a “wound that does not heal” that predisposes for cancer development [2,3]. 

In this review, we focus on intestinal inflammation and the conditions that may contribute to cancer. The maintenance of intestinal homeostasis requires a balance between the intestinal epithelial cells, the immune system, and the gut microbiota [4]. *Drosophila melanogaster* is a simple model where the mechanisms underlying processes like intestinal stem cell proliferation, differentiation and maintenance can be easily studied due to the evolutionarily conserved signaling pathways between *Drosophila* and mammals [5]. Many of these pathways are also activated in the *Drosophila* midgut upon bacterial infection and intestinal epithelium damage, and they are involved in the regeneration of the midgut epithelium. We review the *Drosophila* and the mammalian responses to stress or infection to conclude that epithelially emanating regenerative inflammatory signals similar to those derived from mammalian inflammatory epithelial cells or tumor cells *per se* may directly contribute to cancer initiation, maintenance and progression. 

## 2. Early Lessons from *Drosophila* Systemic Immune Response

### 2.1. Drosophila Systemic Immune Response

**Figure 1:**
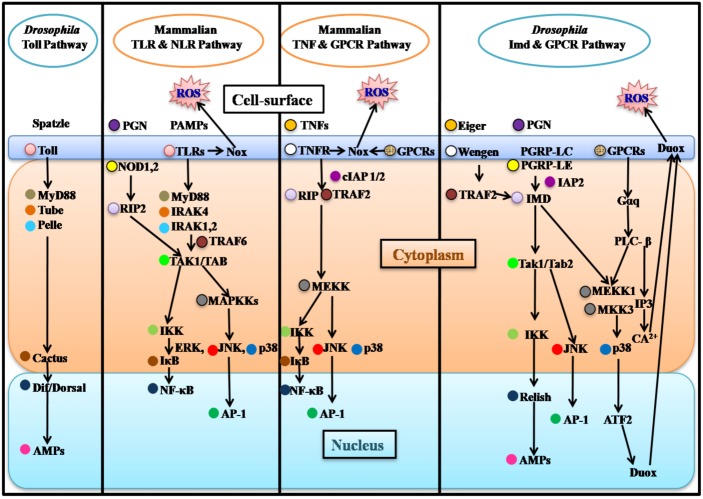
Component conservation among the *Drosophila* and mammalian innate immunity NF-κB pathways. Homologs of the *Drosophila* Toll, Imd, GPCR and Eiger pathways and the mammalian TLR, NLR, TNF and GPCR pathways are marked with circles of the same color at the left of each component. Notice the striking homology of components between species, though some homologs are positioned in different pathways. The subcellular localization of homologous proteins is also conserved. AMPs: Antimicrobial Peptides; Duox: Dual oxidase; GPCRs: G-Protein-Coupled Receptors; NLRs: NOD-like Receptors; Nox: NADPH oxidase; PGN: peptidoglycan; PAMPs: Pathogen-Associated Molecular Patterns; PGRPs: Peptidoglycan Recognition Proteins;ROS: Reactive Oxygen Species; TLR: Toll-like Receptors; TNF: Tumor Necrosis Factor.

During the past 20 years, flies have become an attractive model for studying innate immunity. Numerous studies show that *Drosophila* responds to bacteria, fungi, and viruses *via* the activation of highly conserved pathways e.g., the Imd, Toll, JNK and JAK/STAT pathways, leading to the systemic expression and release of antimicrobial peptides (AMPs) and other factors by the fat body and the hemocytes into the hemolymph [6]. The expression of AMPs is regulated by two critical NF-κΒ pathways, which are activated by bacteria and fungi [6] (Figure 1). The Toll pathway is induced by many bacterial and fungal species, which are recognized by secreted factors, such as GNBP1, PGRP-SA, PGRP-SD and GNBP 3, and which are all able to mediate the proteolytic cleavage and maturation of the Toll receptor-ligand Spätzle (Spz) [7,8]. Toll activation by ligand binding is followed by the recruitment of a receptor–adaptor complex consisting of three death-domain proteins: MyD88, Tube and Pelle [8,9] (Figure 1). Pelle phosphorylates the ΙκB-like protein Cactus, leading to its dissociation from the NF-κΒ-like transcription factor(s) Dorsal and/or Dif, thus allowing them to translocate into the nucleus and activate transcription of AMP genes [8,9]. The second NF-κB pathway that regulates AMP expression in *Drosophila* is immune deficiency (Imd), which is induced by many Gram-negative bacteria through the transmembrane PGRP-LC and the intracellular PGRP-LE peptidoglycan recognition proteins (PGRPs) [7,8] (Figure 1). Interestingly, the Imd signaling pathway involves a Tak1/Tab2 complex, which activates the JNK pathway allowing the nuclear translocation of AP-1 and the IKK complex that regulates the activation of the NF-κB-like protein Relish [7,9]. 

**Figure 2:**
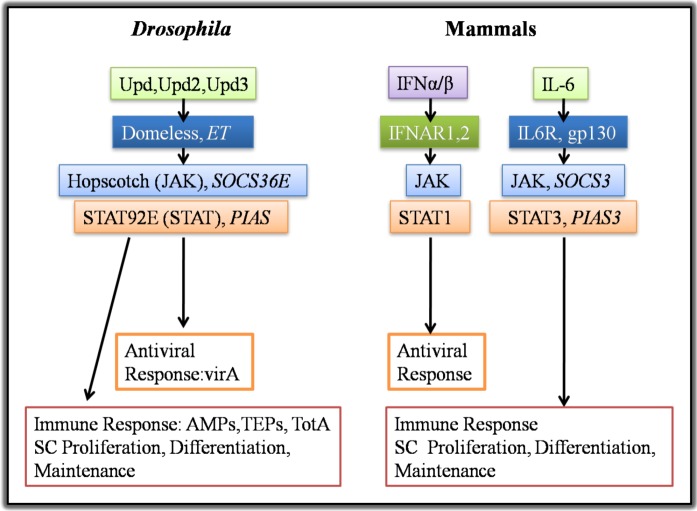
Conservation between the *Drosophila* and the mammalian JAK/STAT pathway and their commonalities in inducing systemic and localized immune response and tissue growth. Activation of the *Drosophila* JAK/STAT pathway by the Upd (Upd, Upd2 and Upd3) cytokines has critical roles in many developmental processes, as well as in immune responses. The JAK/STAT pathways in mammals can be activated by different ligands (e.g., IL-6 and IFNs) that induce distinct signaling cascades. IL-6 binding to its receptor induces innate immunity and tissue growth and maintenance, similarly to the activation of the *Drosophila* JAK/STAT signaling by the Upds. JAK/STAT activation by IFNs promotes antiviral activities. Rectangles of the same colors indicate the homology between the components of *Drosophila* and mammalian JAK/STAT pathway components. Components in italics *i.e.*, the *Eye Transformer (ET)*, *PIAS*, *PIAS3*, *SOCS36E* and *SOCS3* are negative regulators acting at the point of the pathway where they are placed. AMPs: Antimicrobial Peptides; ET: Eye Transformer; JAK: Janus Kinase; IFNAR: Interferon-α/β Receptor; PIAS: Protein Inhibitor of Activated STAT; SC: Stem Cell; STAT: Signal Transducer and Activator of Transcription; SOCS: Suppressor of Cytokine Signaling; TEPs: Thiolester Proteins; Upd: Unpaired.

Furthermore, *Drosophila* infection with bacteria or viruses results in the activation of the JAK/STAT pathway, which is another evolutionarily conserved pathway with multiple roles in development and immunity (Figure 2). Induction of the JAK/STAT pathway following septic injury is mediated by the hemocyte-secreted cytokine Upd3, which is the ligand of the receptor Domeless (Dome) [10]. 

### 2.2. Mammalian Systemic Immune Response and Parallels with Drosophila

In contrast to the indirect recognition mechanism of the fly Toll, the mammalian Toll-like receptors (TLRs), are activated *via* direct binding to pathogen-associated molecules [9]. An oligomer complex similar to *Drosophila* MyD88, Tube and Pelle is utilized during mammalian TLR signaling: IRAK4 and IRAK1 are the mammalian orthologs of the *Drosophila* Tube and Pelle, respectively, while mammalian MyD88 recruits IRAKs and TRAF6 for the activation of the TAK1/TAB complex [7,11]. The downstream signaling is divided into two branches: the first branch emanating from TAK1/TAB stimulates the IKK complex for NF-κB activation and its translocation to the nucleus; and the second branch activates the MAPKKs pathways ERK, JNK and p38. JNK activation induces phosphorylation and nuclear translocation of the transcription factor AP-1 [7,9]. Thus the mammalian TLR pathway has high homology also with the *Drosophila* Imd pathway, downstream of the TAK1/TAB complex [7] (Figure 1). AP-1 comprises a group of sequence-specific transcription factors, which are conventional substrates for JNK and p38 [12]. JNK and p38 belong to the family of mitogen-activated protein kinases (MAPKs), which include the extracellular-signal-regulated kinases (ERKs) and ERK5 subfamilies [12,13]. Moreover, JNK and p38 are mainly activated by pro-inflammatory cytokines in response to stress, while ERK is induced by growth-promoting mitogenic stimuli [13]. TLRs mainly recognize pathogen-associated molecular patterns (PAMPs) in the extracellular environment [14]. However, there is another family of mammalian receptors, known as NOD-like receptors (NLRs), that sense a variety of ligands within the cytoplasm [15]. Similarly to *Drosophila* PGRPs, NOD1 and NOD2 sense peptidoglycan (PGN) fragments (iE-DAP and MDP, respectively) and activate RIP2, which is a serine/threonine kinase homolog of the *Drosophila* Imd [16] (Figure 1). Signaling through RIP2 leads to the activation of NF-κΒ and the production of inflammatory cytokines, while NOD2 signaling pathway additionally leads to the activation of MAPKs [16]. NF-κΒ can also be activated by tumor necrosis factor-α (TNFα) signaling [17]. Interestingly, NF-κΒ activation is regulated by two factors, the *cellular inhibitor of apoptosis 1* and *2* (cIAP1 and cIAP2) [17]. These factors are homologs of the *Drosophila* Imd pathway factor IAP2 (Figure 1). IAP2 is required for the sustained antimicrobial peptide gene expression in the *Drosophila* S2 cells [18].

Mammalian cytokines that belong in the type I interferon (IFN) family induce innate immunity responses against viral infections through STAT1 kinase [19] (Figure 2). On the other hand, the mammalian STAT3 is a main regulator of the differentiation and development of adaptive immunity cells [20,21]. In addition, it mediates the transition from initial innate immune response to infection to a sustained adaptive immune response and has critical roles in inflammation and cancer [22]. It can be activated by IL-6, which is homologous to the *Drosophila* Upd cytokines (Figure 2). IL-6 usually binds to its receptor IL-6R and activates of the signal transducer gp130, *via* the threonine kinase JAK, which subsequently activates the transcription factor STAT3, inducing its nuclear translocation and DNA binding [20]. Moreover, the mammalian IL-6 receptor family members and the gp130 are homologs of the *Drosophila* receptor Domeless and the Eye Transformer (ET), respectively, although the latter is a negative regulator of *Drosophila* JAK/STAT pathway [23]. Pertinent to the negative regulation of the pathway, the *Drosophila* SOCS36E and PIAS have a highly conserved role similar to mammalian SOCS3 and PIAS3, respectively, in inhibiting signal transduction [24,25,26,27] (Figure 2).

## 3. Epithelial Immune Responses of Flies and Mammals

### 3.1. Drosophila Epithelial Immune Responses

In *Drosophila*, ROS and AMPs help the host to fight infection. However, ROS can also damage host cells. To protect the enterocytes from excessive ROS, *immune-regulated catalase* (IRC), is expressed as a response to oxidative stress during gastrointestinal microbial infection [28,29]. The *Drosophila* intestinal immune response depends on whether the invading bacteria are resistant to oxidative stress or not. In the case of ROS-sensitive bacteria, ROS production by Duox fights infection, while detoxification of ROS by IRC protects the host [28,30]. ROS-resistant bacteria may persist in the *Drosophila* intestine and activate the Imd/Relish pathway and subsequent AMP production for the neutralization of bacteria sensitive to AMPs [30]. However, JAK/STAT signaling can also contribute to AMP production [31] [90]. 

Recently another ROS protection gene was shown to protect the host during intestinal bacterial infection in *Drosophila*. The JNK/FOXO pathway regulates the expression of the antioxidant enzyme Peroxiredoxin V (dPrxV) to protect intestinal epithelial cells from oxidative damage, as, for instance, dPrxV mutants exhibit increased lethality during bacterial infection [32]. However, the role of JNK is controversial: Upon aging, oxidative stress leads to abnormal proliferation and differentiation of intestinal stem cells *via* JNK signaling [33], but systemic JNK signaling results in less oxidative damage and lifespan extension [34]. These findings indicate that, on the one hand, JNK signaling induces expression of cytoprotective genes in response to increased stress and oxidative challenge, and, on the other hand, it mediates aberrant stem cell proliferation in the aged enterocytes of *Drosophila* [33,34]. FOXO, a target of JNK, is a transcriptional factor that can influence many biological processes including stress resistance. Under normal conditions it is cytoplasmic in intestinal epithelial cells, while upon intestinal infection it accumulates in the nucleus [32]. Importantly, the expression of dPrxV depends also on the expression of Duox, which generates bactericidal reactive oxygen species (ROS) upon infection [32]. Duox in *Drosophila* is induced by non-peptidoglycan (non-PGN) ligands, which are recognized by G-protein-coupled-receptors (GPCR) and induce the Gaq-PLCβ-IP3-Ca^2+^ pathway (Figure 1), resulting in ROS production in order to maintain balanced gut–microbe interactions [35]. In the absence of infection, the GPCR pathway suppresses the Imd-dependent Duox expression even in the presence of PGN [35]. However, when bacterial infection takes place, PGN induces Duox production *via* PGRP-LC–IMD–MEKK1–p38 signaling and non-PGN stimuli activate MEKK1 through GPCR-Gaq-PLCβ-MEKK1 signaling, resulting in maximal ROS production [35] (Figure 1).

Yet another role for JNK signaling in *Drosophila* is to protect from oxidative stress by activating the expression of several autophagy-related (ATG) genes [36]. The ATG genes are required for the oxidative stress-protection function of the JNK pathway in the *Drosophila* intestinal epithelium [36]. However, only stress-induced autophagy is dependent on JNK signaling [36]. Thus, JNK-mediated expression of ATG genes, can increase the resistance to oxidative stress, but it remains unclear if the same genes also have a role in longevity [36]. The transcription factor(s) downstream of the JNK pathway that mediate the activation of ATG genes are unknown, although FOXO may be one of them [36].

*Drosophila eiger*, the sole homolog of the tumor necrosis factor (TNF) and signaling through its receptor *wengen* (TNF receptor homolog), is suggested to play opposing roles in the fly’s response to infection [37]. This is because there are two TNF receptor-associated factors in *Drosophila*, TRAF1 and 2. The association of each one of them with the receptor *wengen* leads to the activation of different signaling cascades [38]. When TRAF1 associates with *wengen*, apoptosis ensues [38]. On the other hand, the association of TRAF2 with the receptor *wengen* leads to Imd signaling activation and AMP expression [38] (Figure 1).

Strikingly, induction of innate immune response and the Ras oncogene expression in the *Drosophila* hindgut result in the invasion and dissemination of oncogenic hindgut epithelial cells [39]. In the hindgut, bacterial infection induces the Imd pathway, which synergizes with the Ras oncogene to induce the JNK signaling and MMP1 expression. MMP1in turn degrades the extracellular matrix, leading to cell invasion and dissemination [39]. Noticeably, the Imd-JNK-MMP1 pathway in Ras-activated hindgut cells is also activated in immune challenged *Drosophila* hemocyte-like cells [40], suggesting a direct link between epithelially emanating inflammatory signals and cancer cell migration.

### 3.2. Mammalian Epithelial Immune Responses and Parallels with Drosophila

In mammals, TNF receptor (TNFR), Toll-like receptor (TLR), as well as phagocytic activities, activate Nox (NADPH oxidases) enzymes leading to ROS production [35,41] (Figure 1). In the colonic and other human epithelia, the Nox family oxidases, Nox1 and Duox2, are expressed, playing a critical role in chronic inflammation [42,43,44,45]. The human Duox2 is an ortholog of the *Drosophila* Duox [42]. Although the mechanism by which TLRs mediate Nox/Duox-dependent ROS production is not clear [35], TLR4-mediated ROS production is required for the activation of the TRAF6-ASK1-p38 pathway to alert cells of infection [46]. Likewise, in tumor necrosis factor TNFα signaling, ROS generation is needed for the activation of ASK1 by TRAF2 and sustained JNK/p38 activation for the induction of apoptosis [46]. Similarly to *Drosophila*, mammalian G-protein-coupled receptors (GPCR) can induce NADPH oxidases (e.g., Nox) leading to ROS production [47] (Figure 1). Moreover, the mammalian Prx family enzymes are necessary for eliminating ROS in order to protect cells from oxidative cytotoxicity. This process takes place during the activation and the secretory activity of macrophages [48,49]. 

Moreover, Crohn’s disease, which is a type of inflammatory bowel disease (IBD), is associated with autophagy [50,51]. In patients with Crohn’s disease, single-nucleotide polymorphisms (SNPs) were found in autophagy-related genes [50]. Specifically, SNPs have been identified in the autophagy gene ATG16L1, in autophagy-stimulatory GTPase IRGM and in NOD2, which is an intracellular bacterial sensor [50,52]. Normally, NOD2 recognizes the bacterial PGN-derived muramyl-dipeptide (MDP) and recruits ATG16L1, thus inducing autophagy [53]. Importantly, a mutation in the Crohn’s disease susceptibility gene Atg16L1 promotes several hallmarks of the Crohn’s disease upon viral infection [51]. Furthermore, Crohn’s disease is associated with microRNAs that negatively regulate IRGM and autophagy [52,54]. Thus, similar to the induction of autophagy-related (ATG) genes in *Drosophila* upon oxidative stress, the properly set expression of ATG genes in mammals is crucial for epithelial homeostasis.

## 4. Epithelial ISC Responses: Regenerative Inflammation

Cytokines are low-molecular weight polypeptide proteins that are mainly known for their role in immune response and inflammation [55,56]. They are secreted molecules usually acting in the producing (autocrine mode) or adjacent cells (paracrine mode) [57]. Interleukins (IL), interferons (IFN) and tumor necrosis factor (TNF) are the main cytokines [58]. An induced cytokine is able to stimulate the production of many other specific cytokines, in order to create a network of cooperating molecules [55]. However, abnormal levels of cytokines or their receptors results in serious pathologies, such as chronic inflammatory diseases and cancers [56,59]. For example, TNF as well IL-1 and IL-6 are inducible by hypoxia, a hallmark of tumor cells [3]. These cytokines act to suppress cell death, activate stem cells, and promote epithelial proliferation at the site of the injury [60]. Some cytokines may also act as autocrine growth factors to promote the survival of malignant cells [3]. Indeed, IL-6 acts as growth factor for hematological malignancies and IL-1 for gastric carcinoma [3]. 

Growth factors are also polypeptides that usually induce cell growth or proliferation and may have overlapping functions with cytokines in both *Drosophila* and mammals (Table 1). In the following sections, we discuss the growth factor- and cytokine-induced signaling pathways that control the intestinal stem cells maintenance in both mammals and *Drosophila*, thereby emphasizing the overlap in function between growth factors and cytokines during intestinal “regenerative inflammation” and cancer. 

### 4.1. Growth Factors and Cytokines in Intestinal Stem Cell Maintenance

Adult intestinal stem cells (ISC) are present in the *Drosophila* and the mammalian intestine and are responsible for the maintenance of intestinal homeostasis by continuously replacing the intestinal cells, a process mediated by conserved signaling pathways [5,61,62]. The *Drosophila* midgut ISCs are located basally within the intestinal epithelium and give rise to nutrient-absorbing enterocytes and enteroendocrine cells, two differentiated cell types also found in mammals [5]. Before differentiation, ISC are asymmetrically divided into an enteroblast cell and a self-renewing stem cell. The enteroblast differentiates into an enterocyte or enteroendocrine cell depending on the levels of Delta protein, which is the ligand of the Notch signaling pathway [63,64]. In the mammalian intestine, Notch signaling is also required for ISC self-renewal and fate decisions [65]. Notch signaling that promotes ISC proliferation in the mouse intestine, requires cooperation with Wnt, a growth factor also necessary for *Drosophila* ISC maintenance and proliferation [66,67]. 

**Table 1:** Mammalian and *Drosophila* homologous cytokines and growth factors in ISC proliferation and differentiation and EC apoptosis and immune response during homeostasis or infection/stress of the intestine. The first column shows biological processes conserved between *Drosophila* and mammals, while the other two columns represent the corresponding cytokines and growth factors for each species. The homologous components are placed on the same line. The parentheses show critical components of the pertinent signaling pathways in which each cytokine and growth factor are major players. ISC: Intestinal Stem Cells; EC: Enterocytes; PAMPS: Pathogen-Associated Molecular Patterns (e.g., peptidoglycan).

	Mammals	*Drosophila*
**ISC Proliferation/ Maintenance **	Wnt	Wingless
	IL-6 (Stat3)	Upds (JAK/STAT)
	EGF (EGFR-Ras)	Spitz,Keren,Vein (EGFR-Ras1)
**ISC Differentiation**	Ihh	Hh
	BMP	Dpp?
	Wnt	Wingless
	IL-6 (Stat3)	Upds (JAK/STAT)
	EGF (EGFR-Ras)	Spitz,Keren,Vein (EGFR-Ras1)
**EC Apoptosis**	TNF (PAMPs/NF-κB/JNK)	Eiger (PAMPs/NF-κB/JNK)
**EC Immune Response **	TNF (PAMPs/NF-κB/JNK)	Eiger (PAMPs/NF-κB/JNK)
	IL-6 (Stat3)	Upds (JAK/STAT)

Intestinal stem cells also exist in the posterior intestine of *Drosophila*, the hindgut, but they are confined in the *hindgut proliferation zone* (HPZ). Within this zone, ISC self-renewal, proliferation and differentiation, are controlled by Wingless and Hedgehog (Hh) signaling [68]. Specifically, the Hh signaling pathway is required for the transition of ISC from the proliferative to the differentiation state [68]. Similarly, in the crypt epithelium of the mammalian intestine, where ISCs reside, the Wnt and Hh pathway ligands are expressed [68]. Indian Hedgehog (Ihh) induces the formation and proliferation of the mesenchymal cells, which in turn regulate the proliferation and differentiation of nearby ISCs [69]. Wnt signaling is critical for the maintenance of murine intestinal stem cells and progenitors, as it has been shown in mice lacking β-catenin, a positive effector of the Wnt pathway [70]. The blocking of Wnt/β-catenin signaling results in rapid loss of crypts and cell proliferation and terminal differentiation of intestinal stem cells [70]. The opposite phenotypes (increased proliferation, crypt expansion and decreased differentiation) were observed, in experiments with overexpressed β-catenin [70]. Additionally, several signals including the Bone Morphogenetic Protein (BMP) antagonize Wnt signaling to the crypts [61]. Bone Morphogenetic Proteins (BMP) belong to the transforming growth factor beta (TGF-b) superfamily and negatively regulate the ISC proliferation [71]. BMP signaling is maintained by Ihh signals, in order to promote the differentiation of epithelial and mesenchymal cells in the villus [61]. However, in the crypt, there is a production of BMP antagonists by the myofibroblasts, in order to inhibit the BMP signaling, thus maintaining the Wnt activities [61]. 

Loss of function of the Wg pathway in the *Drosophila* midgut does not lead to rapid ISC loss to support the idea that Wg signaling is the main regulator of ISC self-renewal and differentiation, as in the mammalian model [72]. Instead, the Wg, JAK/STAT and EGFR/Ras/Erk signaling pathways cooperate to maintain ISCs [73]. Thus, the simultaneous disruption of all three of them results in complete elimination of ISC in a short time, while disruption of a single one can be replaced by over-activation of one of the other two [73]. 

Wg and the EGFR signaling pathway ligand Vein are expressed in the *Drosophila* visceral muscle, which acts as a stem cell niche [72,73,74]. JAK/STAT pathway is induced by ligands emanating from the visceral muscle [73] or the intestinal epithelium cells [75]. On the other hand, Notch signaling represses transcriptionally JAK/STAT signaling ligand unpaired (upd) [76]. Conversely, JAK/STAT antagonize Notch signaling during enteroblast fate decision [77]. High levels of JAK/STAT signaling leads to differentiation into enteroendocrine cells, while low levels of its activation, preferentially lead to differentiation into enterocytes [77].

Three of the pathways that regulate ISC maintenance in *Drosophila*, are also induced by niche signals that are provided by the mammalian Paneth cells, including EGF (EGFR ligand), Wnt3 (Wnt ligand) and Dll4 (Notch ligand) [78]. Thus, Paneth cells have been characterized as “multifunctional guardians” of the mammalian intestinal stem cells [78]. Nevertheless, sub-epithelial myofibroblasts are also proposed to maintain mouse ISCs [79]. The overall process is strikingly similar to the *Drosophila* cytokines and growth factors emanating from both the epithelium [75] and the visceral muscle [73].

Interestingly, the *Drosophila* midgut and the mammalian intestine share similarities in the symmetry of ISC divisions. Recent studies, suggest that 2 out of 10 ISC divisions in the *Drosophila* midgut, are symmetric to balance for the occasional loss of ISCs [80,81]. This is similar to the Lgr5+ mammalian ISCs, which compensate for stem cell loss by symmetric division [82]. 

### 4.2. Intestinal Epithelium Regeneration and Cancer-Promoting Inflammation

**Figure 3:**
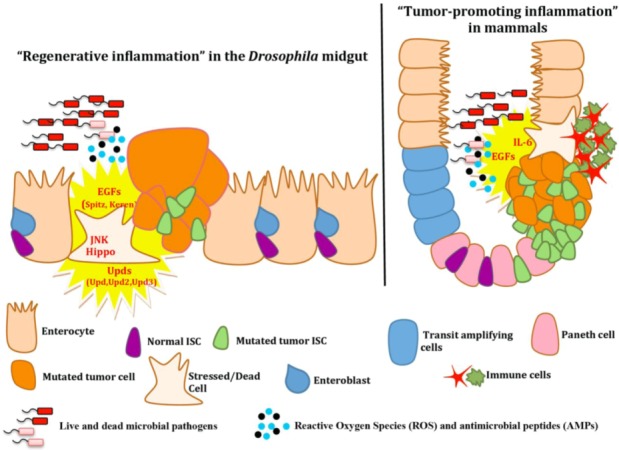
“Regenerative inflammation” in the *Drosophila* midgut resembles but also differs from “tumor-promoting inflammation” in mammals. Upon intestinal bacterial infection in *Drosophila*, growth factors (EGFs) and cytokines (Upds: IL-6-like cytokines) are secreted by the damaged epithelial cells and activate ISC proliferation and differentiation in order to regenerate the damaged midgut epithelium. Homologs of these growth factors and cytokines have also been observed in mammalian tumor-initiating epithelial cells. In contrast to the *Drosophila*, “regenerative inflammation”, which is directly induced by epithelially emanating signals, the mammalian “tumor-promoting inflammation” includes the infiltration of blood cells at the place of the damage and ISC proliferation. Reactive oxygen species (ROS) are also activated in both species and have opposing roles upon damage: they help the host to fight the infection, but they may also contribute to mutagenesis and tumor formation.

The *Drosophila* midgut epithelium is a dynamic tissue, capable of regenerating the whole intestinal epithelium when damaged or infected by expressing growth factors and signals that promote ISC proliferation and differentiation. Over the years, many studies indicate inflammation and immune signals as enabling characteristics of cancer, although the connection between inflammation and cancer is not fully elucidated [2]. In mammals, stressed or dying cells due to infection promote inflammation by the activation of different types of immune cells e.g., macrophages, neutrophils, T-cells and B-cells, which in turn activate a variety of tumor-promoting inflammatory cytokines (Figure 3) [60]. Extensive chronic tissue damage and cell death perpetuates inflammation and regeneration by an increase in stem cell proliferation, in addition to a higher probability in harboring oncogenic mutations [60]. Inflammatory cytokines are also expressed by cancer cells, which in turn recruit immune cells leading to tumor-associated inflammation [60]. Most importantly, infiltrating blood cells in the tumor microenvironment are traditionally believed to be the instigators of tumor-promoting inflammation [2,3].

In contrast to mammals, the *Drosophila* intestine does not appear to be infiltrated by hemocytes (*Drosophila* phagocytes [83]) in response to infection. Although phagocytosis by hemocytes is crucial in fighting intestinal infections when bacteria escape from the intestine into the hemolymph e.g., upon infection with the entomopathogenic bacterium *Serratia marcescens* [84], this process does not lead to the infiltration of hemocytes into the epithelium [83,84]. On the contrary, *Drosophila* intestinal epithelium-emanating pro-inflammatory signals (cytokines and growth factors) can directly promote ISC proliferation and differentiation and regenerate the damaged epithelium. This “regenerative inflammation” is a dynamic process mainly controlled by at least four evolutionary conserved signaling pathways (Figure 3). Two of them are the JNK and the Hippo signaling pathways, which are activated as a consequence of intestinal epithelium infection in order to firstly induce the production of cytoprotective genes (JNK) or in damaged or stressed cells to induce ISC proliferation and regeneration (JNK and Hippo) [85]. JNK and Hippo signaling promotes the expression of IL-6-like pro-inflammatory cytokines *unpaired (upd)*, *unpaired 2 (upd2)* and *unpaired 3(upd3*) by the damaged midgut epithelium cells, as well the secretion of EGFR signaling pathway-ligands promoting over-proliferation of ISC and intestinal hyperplasia [5,85,87]. Moreover, upon bacterial infection Upd3, which is released by the enterocytes, seems to have an additive effect with Upd2 in the induction of epithelial regeneration [75].

The other key player in *Drosophila* midgut epithelium regeneration is the EGFR/Ras/MAPK pathway. The epidermal growth factor receptor (EGFR) is activated by three EGF ligands: Spitz and Keren (produced within the intestinal epithelium) and Vein, which is produced by the visceral muscles [86,88]. Induction of Vein in the visceral muscles requires the activation of the JAK/STAT pathway by the Upd3 cytokine, which is expressed by damaged enterocytes [86]. Upd3 can also induce Spitz in enteroblasts [88]. Independently of their source, these EGFR pathway ligands induce ISC proliferation and midgut hyperplasia [85]. 

More recent studies show that Wg is another damage-inducible pathway, which is required for ISC proliferation during *Drosophila* midgut regeneration [89]. Wg is also an important target of the JNK signaling. The activation of JNK in ECs upon intestinal damage or stress, results in the secretion of Wg by the EBs and the activation of Wg signaling, which in turn upregulates Myc in the ISCs and leads to their proliferation [89]. Importantly, regeneration, but not ISC self-renewal, requires Wg expression in the midgut enterocytes [89].

Various bacterial pathogens can activate the aforementioned conserved signaling pathways to induce regeneration of the damaged or stressed *Drosophila* intestine. For example, *Drosophila* infection with high doses of *Pseudomonas entomophila* induces epithelium renewal while even higher concentration of the same bacterium leads to ISC loss [90]. The JNK and JAK-STAT pathways are induced in the gut cells upon infection with *P. entomophila*, *Erwinia carotovora carotovora 15* (Ecc15), *Serratia mascescens* and *Pseudomonas aeruginosa* [90]. Interestingly, when bacterial infection is combined with low cytologically innocuous expression levels of an oncogenic form of Ras1 gene (ortholog of the mammalian K-Ras) in the midgut ISCs and progenitors, intestinal dysplasia ensues. This is due to a synergism between the bacterial infection-induced JNK and the Ras oncogene [40,91]. In this model, the virulent strain (PA14) of the human opportunistic pathogen *P. aeruginosa* damages and induces regeneration of the epithelium *via* JNK signaling [91]. In contrast, the avirulent (CF5) *P. aeruginosa* strain causes no damage in the midgut epithelial cells [91]. The virulence factor *pyocyanin* secreted by the virulent but not by the avirulent *P. aeruginosa* contributes to the ISC over-proliferation during infection [91].

The capability of pathogenic bacteria to induce cancer initiation and progression was also examined in mammalian models. Recently, *Escherichia coli* was shown to induce intestinal tumorigenesis and inflammation in mice. *E. coli* strain NC101 harboring a polyketide synthase (pks), which is a DNA-damaging toxin, known as colibactin, is required for the progression of colorectal cancer (CRC) in carcinogen-treated interleukin-10 deficient mice [92]. Importantly, NC101 was detected in 40% of inflammatory bowel disease (IBD) patients and in almost 70% of CRC patients, indicating pks as a potential tumor-promoting factor [92]. This and other studies in mammals show that apart from intestinal damage and inflammation, the genotoxicity are properties of some bacteria that may promote human CRC. Consistently, a hypothetical model proposed by Ben-Neriah and Karin links ROS and nitric oxide (NO) production with mutagenesis of critical genes in the intestinal stem cells, such as the gene encoding adenomatosis polyposis coli (APC), resulting in an adenoma growth and colorectal tumor generation [93].

Noticeably, the majority of the signaling pathways that contribute to the *Drosophila* regenerative inflammation may also contribute to tumor initiation and progression in mammals. The mammalian JAK/STAT pathway signaling requires NF-κB activation for the production of pro-inflammatory cytokines and growth factors during colitis-associated cancer (CAC) [94]. Interleukin-6 (IL-6) is one NF-kB-dependent cytokine, which induces the oncogenic transcription factor STAT3 in order to promote proliferation and survival of tumor-initiating intestinal epithelial cells, thus contributing to CAC tumorigenesis in mice [94]. Importantly, the cytokine IL-6 acts not only in epithelial but also in immune cells and is produced by the lamina propria, a layer of connective tissue, which does not exist in the *Drosophila* intestine [5,94]. Lamina propria is located under the intestinal epithelium and, together with the epithelium, houses many immune cells e.g., macrophages, dendritic cells and B-cells [5]. The absence of this layer in the *Drosophila* intestine correlates with the absence of immune cells in the *Drosophila* intestine, but further studies are required to clarify if *Drosophila* hemocytes play any role in intestinal inflammation.

Regardless, two ligands of the mammalian EGFR pathway, amphiregulin (AREG) and epiregulin (EREG), are induced by the pro-inflammatory cytokines IL-1β and TNF-a in inflamed colonic mucosa and in adenomas and carcinomas of human colon, but not in normal colonic mucosa [22]. Importantly, in patients with ulcerative colitis and Crohn’s disease, the epithelial cells rather than the mesenchymal cells, exhibit high expression of amphiregulin and epiregulin [22]. Thus an auxiliary mechanism of inflammation, similar to that observed in the *Drosophila* intestine may exist in the human intestine, where pro-inflammatory signals and growth factors emerging from the inflamed colonic epithelial cells and the tumors may act directly in a paracrine manner to facilitate intestinal stem cell proliferation and tumor progression. Furthermore, epithelial cells surrounding colon cancer stem cells secrete a hepatocyte growth factor (HGF) and maintain high Wnt activity in colon cancer stem cells, but also induce the activation of Wnt in differentiated cancer cells [95]. Pattern recognition receptors e.g., Toll-like receptors (TLR) are also activated in epithelial cells during tumorigenesis by oxidative stress, bacterial products and tissue damage [96]. These findings suggest that the epithelial microenvironment may contribute significantly to the propagation of the colon cancer cells [95].

In infected *Drosophila*, the Toll pathway activity appears to limited to the systemic immune response *i.e.*, in the fat body and hemocytes, where Toll acts as an immune sensor [97]. In contrast, the Imd pathway is activated both systemically and in the midgut and hindgut epithelium [97]. In the infected midgut epithelium, Imd acts in a p38-dependent manner to regulate ROS production and in a Relish-dependent manner for the local expression of AMPs [29,97,98]. Whether NF-κB-mediated immune response in flies is linked to the regeneration process is still an open question.

## 5. Other Frontiers in *Drosophila* Inflammation

### 5.1. Organ Communication: Inflammatory Signal Cross-Talk between Different Organs

*Drosophila* larvae hemocytes communicate with the fat body upon septic injury and oral infection [99]. In addition, cytokines control AMP expression in the larval fat body in a process that strongly resembles the mammalian response to bacterial infection [99]. This is mediated by the Toll ligand Spätzle, a cytokine secreted by the hemocytes [96]. Thus, Spätzle’s knockdown in the larvae hemocytes blockes the expression of the AMP gene *drosomycin*, in the fat body [96]. Furthermore, a lysosomal protein Psidin has a dual role in the immune response upon infection in the detection of the bacteria by the hemocytes, and in the activation of the AMP gene *defencin* in the fat body [100]. Moreover, *Drosophila* Upd3 expression in hemocytes induces JAK/STAT signaling in the fat body, in order to activate the expression of *totA* peptide, which is suggested to be a general stress-response factor [10,101]. Interestingly, *totA* also requires the Imd/Relish signaling, which is activated in response to many Gram-negative bacteria, in the fat body cells [10]. These findings suggest that, apart from their phagocytic activities, the hemocytes of the *Drosophila* larvae also act through acytokine-based regulatory signal, similar to mammalian innate immune response, which comprises the release of cytokines and chemokines by activated immune cells (e.g., macrophages) upon bacterial infection [99].

In a tumor model (*Ras^V12^*/*scrib^−/−^)* of eye–antennal imaginal discs of *Drosophila*, larval hemocyte number increases through the activation of the JAK/STAT pathway [102]. The STAT transcription factor is highly induced in these tumors, and in the circulating hemocytes, but not in the wild-type larvae [102]. JAK/STAT is also activated in mechanically wounded larvae discs. Furthermore, the local activation of the JNK pathway induces the expression of JAK/STAT ligands (unpaired cytokines) in both tumors and wounds [102]. This model suggests that JNK signaling in the damaged tissue induces the expression of Upd ligands and the subsequent activation of the JAK/STAT pathway in the hemocytes and the fat body, resulting in additional cytokine expression, and as a consequence an increase in the hemocytes number [102]. 

Organ-to-organ communication also occurs between *Drosophila* gut, hemocytes and the fat body, during the larval innate immune response [103,104]. Upon bacterial infection nitric oxide (NO) is produced in the gut, while the hemocytes, which are activated *via* the NO-depended signal, function as an intermediary in order to pass the signal to the fat body, resulting in the production of the AMP *Diptericin* [103,104]. Activation of the Rel/NF-κΒ pathway in the fat body cells is required for AMP production [103,104]. 

### 5.2. Intestinal Microbiota and Inflammation

The characterization of *Drosophila* gut microbiota and the capability of some bacteria to produce cancer-related phenotypes in synergy with the genetic predisposition in *Drosophila* intestine, as well the high conservation of the mechanisms and the signaling pathways that regulate ISC maintenance and innate immunity between *Drosophila* and mammals, have made *Drosophila* an attractive model for understanding the interactions occurring between the microbiota and the human gut, as well their potential role in gut pathogenesis, inflammation and cancer. 

There are usually about 1 to 20 different species of bacteria in the *Drosophila* gut, while in the mammalian intestine, there are at least hundreds of different species. *Drosophila* microbiota is therefore much simpler [105,106]. In *Drosophila*, only a few aerotolerant bacteria species are found e.g., *Lactobacillus* species, while the strictly anaerobic species such as *Bacteroidetes* that are abundant in the human flora, are absent the *Drosophila* intestine [105]. *Lactobacillus plantarum* and *Enterococcus faecalis* were recently tested for their ability to colonize germ-free *Drosophila*. Although both of them can colonize young larvae, only *L. plantarum* is considered innocuous or beneficial and remains associated with *Drosophila* long after initial colonization [107]. Indeed, several strains of *L. plantarum* stimulate larval development upon nutrient scarcity and adults emerge faster than in the germ-free animals [107]. *E. faecalis* is another common colonizer of the human bowel [108]. Nevertheless, virulent *E. faecalis* strains produce cytolysin, which has a dual role both as a toxin and a bacteriocin [108]. Flies feeding on a virulent, cytolysin toxin-expressing *E. faecalis* strain exhibit significantly increased lethality [108]. Additionally, a virulence determinant possessing homology to many human pathogenic bacteria, termed KerV, is crucial for the pathogenesity of several bacterial species [109]. On of them is *P. aeruginosa*, a principal agent of lethal infections in cystic fibrosis, severely wounded and cancer patients [110]. Two more are *Vibrio cholera*, the etiological agent of cholera and *Yersinia pseudotuberculosis*, a gastrointestinal pathogen, both of which require the kerV gene to exert full virulence upon introduction in the adult *Drosophila* intestine [109]. In addition, host metabolism can be modified by commensal bacteria, such as *Acetobacter pomorum* in *Drosophila*. *A. pomorum* modulates insulin/insulin-like growth factor signaling, which in turn affects the developmental rate, the metabolism and the intestinal stem cell activity [111].

Host genes also shape intestinal microbiota. In *Drosophila* gut epithelia, the caudal protein is required in order to repress NF-κΒ-dependent AMP expression and, in turn, maintain a balanced flora community [98]. In caudal knockdown flies, there is an overexpression of AMPs, which results in an unbalanced community structure (dysbiosis) and a subsequent elevated gut apoptosis and host death [98]. Inflammatory bowel diseases also involve apoptosis of human intestinal cells. Thus perturbations in the intestinal NF-κΒ pathway may be relevant to the etiology of intestinal pathogenesis in both mammals and flies [112]. Furthermore, it was demonstrated that dual oxidase (Duox) activity in the fly gut is controlled by multiple Duox-regulatory signaling pathways, which “fine-tuned” ROS production depending on the type of gut–microbe interactions [113]. That is, negative regulation of Duox occurs in response to colonization with commensal microbes while, if infectious microbes colonize the gut, there is a positive regulation of Duox [113]. 

In conclusion, gut microbiota provide protective, metabolic and nutritional signals and help the host to ward off harmful microbes that elicit intestinal damage and concomitant inflammation [114]. Therefore, in order to maintain a healthy intestinal epithelium, a harmonious coordination of the gut microbiota, immune and stem cell responses, and environmental factors such as diet, is required [90]. If the delicate balance between these factors breaks, inflammatory diseases may develop. 

## 6. Conclusions

During the last 20 years, *Drosophila* has provided invaluable insights in the field of innate immunity. It helped tremendously to decipher the mammalian innate immune responses and for that, a *Drosophila* scientist, Jules Hoffman, was co-awarded the Nobel Prize in Physiology or Medicine in 2011. While the conservation of innate immune responses between insects and mammals is now literally textbook knowledge, cytokine and growth factor signaling pathways have been recently shown to induce epithelial immunity and regeneration that facilitates cancer-related phenotypes. Contrary to mammals, this “regenerative inflammation” does not require *Drosophila* hemocytes in order to induce ISCs and predispose for tumor formation. This apparent discrepancy might be due to physiologic differences between flies and mammals e.g., the lack of lamina propria in flies where mammalian blood cells accumulate upon inflammation. Nevertheless, studies on *Drosophila* “regenerative inflammation” might help to decipher the role of epithelially emanating cytokines and growth factors in ISC induction in the absence of blood cell infiltration. Because chronic inflammation, while irrefutably a major driver of carcinogenesis, manifests itself in only a subset of cancers [3], we believe that even in the absence of blood cell infiltration, increased intestinal regeneration propelled directly from epithelial cytokines and growth factors might be the instigator of a more broadly defined inflammation-driven carcinogenesis.
